# Optimal drug regimens for improving ALP biochemical levels in patients with primary biliary cholangitis refractory to UDCA: a systematic review and Bayesian network meta-analysis

**DOI:** 10.1186/s13643-024-02460-0

**Published:** 2024-01-29

**Authors:** Wei Lin, Jun-xi Wang, Yi-juan Liu

**Affiliations:** 1 Department of Endoscopy Center, National Regional Medical Center, Binhai Campus of the First Affiliated Hospital, Fujian Medical University, Fuzhou, 350212 China; 2 Department of Gastroenterology, National Regional Medical Center, Binhai Campus of the First Affiliated Hospital, Fujian Medical University, Fuzhou, 350212 China

**Keywords:** Refractory primary biliary cholangitis, Ursodeoxycholic acid, Alkaline phosphatase

## Abstract

**Background:**

Up to 40% of UDCA-treated patients do not have an adequate clinical response. Farnesoid X receptor agonists, peroxisome proliferator-activated receptor agonists, and fibroblast growth factor 19 analogs were developed as adjunctive therapy. The aim of this network meta-analysis was to compare the efficacy of these drugs as add-on therapy for patients with primary biliary cholangitis (PBC) refractory to UDCA in improving ALP levels.

**Methods:**

We searched PubMed, Embase, Web of Science, and the Cochrane Library for eligible studies until 1 December 2023. Randomized controlled trials, cohort studies, and case–control studies comparing the efficacy of different combination treatments and UDCA monotherapy in UDCA-refractory PBC patients were included in the analysis. Cumulative probability was used to rank the included treatments.

**Results:**

A total of 23 articles were eligible for our network meta-analysis. In terms of improving ALP levels, In terms of improving ALP biochemical levels, bezafibrate combined with UDCA (MD 104.49, 95% CI 60.41, 161.92), fenofibrate combined with UDCA (MD 87.81, 95% CI (52.34, 129.79), OCA combined with UDCA (MD 65.21, 95% CI 8.99, 121.80), seladelpar combined with UDCA (MD 117.39, 95% CI 19.97, 213.95), elafibranor combined with UDCA (MD 140.73, 95% CI 74.34, 209.98), saroglitazar combined with UDCA (MD 132.09, 95% CI 13.99, 247.04) was more effective than UDCA monotherapy. Elafibranor in combination with UDCA was the most likely (32%) to be the optimal drug regimen.

**Conclusion:**

As second-line therapy for UDCA-refractory PBC, PPAR agonists were more effective than any other drugs with other mechanisms in improving ALP biochemical levels, with elafibranor being the best.

## Introduction

Primary biliary cholangitis (PBC) is a chronic inflammatory autoimmune-mediated liver disease that is characterized by primary progressive destruction of the small intrahepatic bile ducts, impaired biliary secretion, and hepatocellular retention of toxic endogenous bile acids [1]. The incidence of PBC ranges from 0.33 to 5.8 per 100,000 inhabitants/year and prevalence rates range from 1.91 to 40.2 per 100,000 inhabitants, middle-aged or elderly women are more affected with a female-to-male ratio of 9 to 1 [2]. Ursodeoxycholic acid (UDCA) has been regarded as the standard treatment for PBC, resulting in improved liver tests, alleviation of symptoms, and increased transplant-free survival [3]. However, approximately 30–40% of UDCA-treated patients do not have an adequate clinical response, and such patients have significantly lower transplant-free survival rates as compared to responsive patients [4–6]. Persistent elevated ALP levels mean an increased risk of end-stage liver disease and death [7]. Accordingly, there is a significant medical need for new therapies for the treatment of refractory primary biliary cholangitis. Novel farnesoid X receptor agonists (FXR), peroxisome proliferator-activated receptor (PPAR) agonists, and fibroblast growth factor 19 (FGF19) analogs are in development and may act as second-line therapy for primary biliary cholangitis refractory to UDCA. The mechanism of farnesoid X receptor (FXR) includes inhibition of bile salt synthesis from cholesterol and enhancement of bile acid conjugation. Obeticholic acid (OCA), the naturally occurring ligand of the farnesoid X receptor (FXR), which has been approved in May 2017 by the FDA for patients with an inadequate response to UDCA [5]. Fibrates have the potential ability to decrease bile acid synthesis and bile acid-related hepatic inflammation mediated through the peroxisome proliferator-activated receptor signaling axis. Fenofibrate, a PPAR-α agonist [8–12], bezafibrate, a panPPAR agonist [13–15], elafibranor, a dual PPAR-α and PPAR-δ agonist [16], saroglitazar, a dual PPAR-α and PPAR-γ agonist [17, 18], seladelpar (MBX-8025), a selective PPAR-δ agonist [19–21], have shown satisfying effect in decreasing markers of cholestasis and improving liver function in primary biliary cholangitis patients with incomplete UDCA response. Aldafermin (NGM282) [22] is an analog of FGF19 being evaluated for the treatment of PBC. FGF19 is an endocrine hormone induced in the gut by activation of farnesoid X receptor (FXR), which can inhibit bile acid synthesis through inhibition of CYP7A1 [23]. Several traditional meta-analyses [11, 14, 15] manifested that fenofibrate and bezafibrate are effective adjunctive therapies in the treatment of primary biliary cirrhosis. some single or combined therapies about obeticholic acid [24–26], aldafermin [27], seladelpar [28, 29], elafibranor [16], saroglitazar [18], and budesonide [30] in the treatment of UDCA-refractory PBC patients have been studied by randomized controlled trials (RCTs). However, which treatment regime is optimal for patients with UDCA-refractory PBC remains controversial. Therefore, we conducted a network meta-analysis to compare the efficacy of multiple interventions in the management of PBC. The primary objective of this systematic review with network meta-analysis was to provide evidence-based suggestions for clinical decision-making. Ethical approval was not necessary, because this is a meta-analysis.

### Systematic literature search

We searched four electronic databases (PubMed, Embase, Web of Science, and the Cochrane Library) up to 1 December 2023 for randomized controlled trials, cohort studies, and case–control studies without any language restrictions. The following search terms were applied: ‘‘PBC’’ or ‘‘primary biliary cholangitis’’ or ‘‘primary biliary cirrhosis’’ or ‘‘UDCA’’ or ‘‘Ursodeoxycholic Acid’’ or “bezafibrate’’ or ‘‘fenofibrate’’ or “budesonide” or “seladelpar’’ or “MBX-8025’’ or “elafibranor’’ or “saroglitazar’’ or “peroxisome proliferator-activated receptor” or “PPARs’’ or “OCA’’ or “obeticholic acid’’ or “FXR” or “farnesoid X receptor agonist” or “aldafermin’’ or “NGM282’’ or “FGF19’’. All potential studies were searched and identified by two investigators independently. Additional studies in the reference lists of all identified publications, including relevant meta-analyses and systematic reviews, were also searched to avoid omitting relevant publications.

### Selection criteria

Eligible articles were required to fulfill the following inclusion criteria: (1) randomized controlled trials or cohort studies or case–control studies with clear outcomes and raw data that can be obtained; (2) PBC patients with a suboptimal response to UDCA monotherapy; (3) included at least one combined therapy arm (UDCA plus fenofibrate or bezafibrate or OCA or budesonide or seladelpar or elafibranor or saroglitazar or aldafermin) with or without a UDCA monotherapy arm. (4) having been performed on adults. The exclusion criteria were as follows: (1) case reports, letters, conference papers, systematic reviews, and meta-analyses were excluded; (2) studies performed on animals or children; (3) whether patients with primary biliary cholangitis respond well to UDCA monotherapy is not explicitly mentioned; (4) combined therapy or monotherapy was compared with placebo but not UDCA monotherapy; (5) raw data could not be extracted to obtain pooled results; (6) duplicated articles; (7) other diseases.

### Data extraction strategy

Trials included multiple treatment groups with different doses, each of which was included in the traditional pairwise meta-analysis while only the optimal dose was included in the network meta-analysis except for saroglitazar. Due to the higher incidence of elevated liver enzymes observed with the 4 mg dose, saroglitazar at doses of 2 mg was included. Nevens et al. [25] divided obeticholic acid into the 5–10 mg group (at an initial dose of 5 mg with adjustment to 10 mg) and the 10 mg group (at a dose of 10 mg), to minimize the heterogeneity, we only included the 10 mg group in our network meta-analysis. The optimal dose included in the network meta-analysis is summarized as follows: OCA 10 mg, aldafermin 3 mg, saroglitazar 2 mg, elafibranor 80 mg, seladelpar 10 mg.

We found that one or more of the cells were equal to 0 in the calculation of OR of adverse event rates when we attempted to perform network meta-analysis of safety profile in different arms, this clearly could bring large heterogeneity to make our conclusions unreliable so we abandoned this scheme.

To ensure the accuracy, two independent reviewers performed data abstraction and resolved the discrepancies by consensus with the third reviewer. Any data discrepancy at any stage was resolved by referring to the original article. Data extracted included the name of the first author, year of publication, study design, study size, drug dosage, duration of study, and ALP levels before and after treatment.

### Quality assessment

**Fig. 1:**
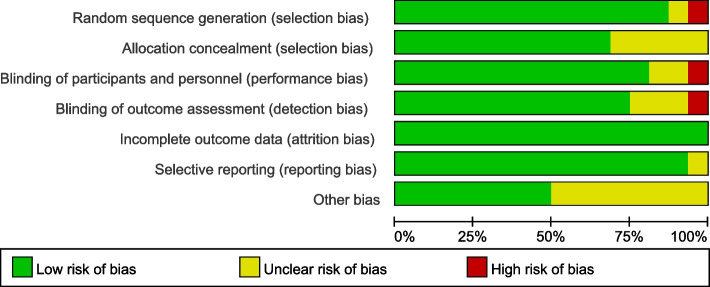
Risk of bias graph

The risk of bias assessment was performed by two independent reviewers using the Cochrane Risk of Bias Tool with ReviewManager (RevMan) (Version 5.3, Cochrane Collaboration). Each study was evaluated based on random sequence generation, allocation concealment, blinding of participants and personnel, blinding of outcome assessment, and reporting. The quality of observational studies was assessed using the ROBINS-I tool [31]. The quality of the included studies was overall high, as shown in the graph of the risk of bias (Fig. 1) and the risk of bias assessment with ROBINS-I (Table 1).

**Table 1:** Risk of bias assessment with ROBINS-I

Study	1	2	3	4	5	6	7	Overall
Cheung 2016	M	L	L	L	NI	L	M	M
Liberopoulos 2010	M	L	L	S	M	L	L	S
Wang 2022	M	L	L	NI	L	L	M	M
Dohmen 2013	M	L	L	S	M	L	L	S
Ding 2022	M	L	L	NI	L	L	M	M
Ding 2023	M	L	L	L	NI	L	M	M

Risk of bias options are *L* Low, *M* Moderate, *S* Serious, *C* Critical, and *NI* No information

1 bias due to confounding, 2 bias in the selection of participants into the study, 3 bias in classification of interventions, 4 bias due to deviations from intended interventions, 5 bias due to missing data, 6 bias in the measurement of outcomes, 7 bias in the selection of the reported result

The seven bias domains are individually assessed for each study

### Statistical analyses

A network meta-analysis was used to indirectly compare the effects of all treatment regimens for primary biliary cholangitis refractory to UDCA monotherapy. Using Stata (version 17.0) software to construct network diagrams, lines connect the interventions that have been studied in head-to-head (direct) comparisons in eligible controlled trials. Different nodes referred to different interventions accordingly, the width of the lines is proportional to the number of trials comparing each pair of treatments, and the size of each node is proportional to the number of participants (sample size). Pair-wise meta-analyses were performed by theDerSimonian–Laird random-effects model of the (ADDIS) software (version 1.16.8) to calculate the pooled estimates of mean difference (MD) and 95% confidence interval (CI). Heterogeneity between studies was assessed by the *Q* test and *I*^2^, *I*^2^ values < 25%, 25–75%, and > 75% represent mild, moderate, and severe heterogeneity, respectively [32]. The network meta-analysis was performed using a Bayesian random-effects model, using the Markov chain Monte Carlo simulation implemented through the Aggregate Data Drug Information System (ADDIS) software (version 1.16.8). All data in the current study was analyzed using the ADDIS software, and presented with mean difference (MD) and 95% credibility interval (CrI). We used a consistency model if there is no relevant inconsistency in the evidence. The pooled mean differences (MD) from the network meta-analysis were compared with the corresponding MD from the pairwise random effects meta-analysis of direct comparisons to assess whether there was inconsistency between direct and indirect comparisons. Node-splitting analysis was also performed to prove that no statistical inconsistency existed when *P* > 0.05 [33]. If the network meta-analysis lacks a complex evidence structure (no closed loops), a consistency model can also be used; in other cases, an inconsistency model must be applied [34]. The models were based on 50,000 iterations for each 4 chains with a burn-in period of the first 20,000 iterations. The convergence degree of the model was estimated using the Brooks-Gelman-Rubin method [35], and presented with the potential scale reduction factor (PSRF). The more PSRF approximated to 1, the better convergence was obtained. A relevant rank probability plot could present the best therapeutic measure. We assumed that rank 1 is the most effective and Rank N is the least effective.

For statistical analysis, biochemical markers of interest were treated as continuous variables with mean and standard deviation. In several included studies, data were presented in terms of medians and quartiles 1st–3rd/range; in these cases, valid formulas were used for data estimation and conversion [36–38]. Measurements were converted to reflect the same units across studies: IU/L for alkaline phosphatase.

## Results

**Fig. 2:**
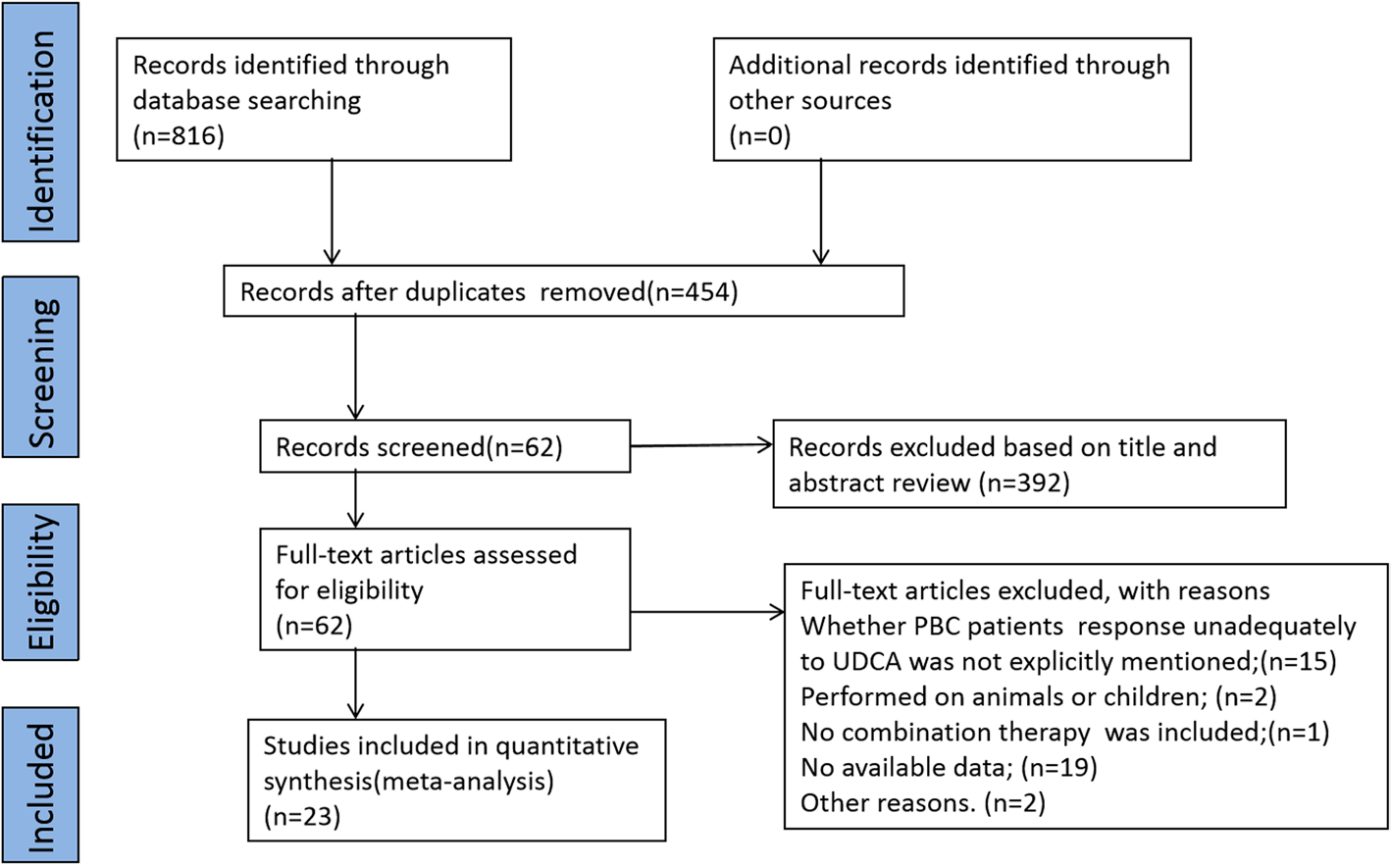
Flow chart of included studies

**Fig. 3:**
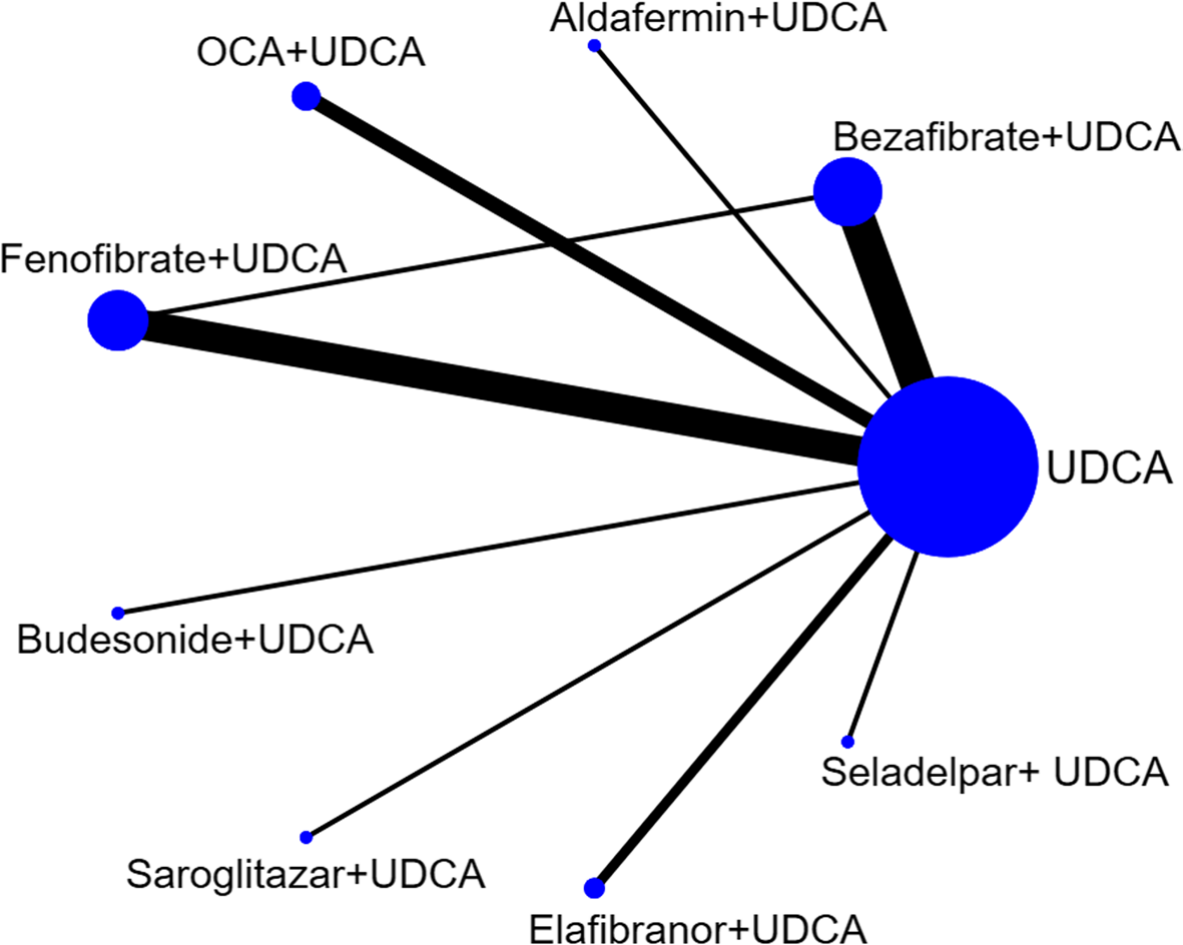
Network of interventional treatments included in meta-analysis

The literature search identified 816 citations and 62 studies underwent full manuscript review. After the full-text screening,39 articles were excluded, leaving a total of 23 studies involving 1734 participants eligible for the systematic review (Fig. 2). The description of the trial characteristics is given in Table 2. UDCA plus bezafibrate was compared with UDCA monotherapy in seven trials [13, 39–44]. UDCA plus fenofibrate was compared with UDCA monotherapy in six trials [10, 45–49]. UDCA plus budesonide was compared with UDCA monotherapy in one trial [30]. UDCA plus bezafibrate was compared with UDCA plus fenofibrate in one trial [50]. UDCA plus OCA was compared with UDCA monotherapy in three trials [24, 25, 51]. One trial [26] compared OCA monotherapy with placebo was excluded. UDCA plus aldafermin was compared with UDCA monotherapy in one trial [27]. UDCA plus elafibranor was compared with UDCA monotherapy in two trials [16, 52]. UDCA plus saroglitazar was compared with UDCA monotherapy in one trial [18]. A phase II randomized double-blind controlled study published by Jones et al. [19] manifested that seladelpar normalized ALP levels in patients who completed 12 weeks of treatment. However, treatment was associated with grade 3 increases in aminotransferases, which amplified that the effects of seladelpar should be explored at lower doses and the study was stopped early. Another phase II randomized double-blind controlled study subsequently published by Bowlus et al. [29] manifested that seladelpar showed a significant dose-dependent improvement in ALP levels in PBC patients with poor UDCA response, but the study did not set a control group, so it could not be included in the network meta for statistical analysis. Our study only included one appropriate study [53] about seladelpar in the analysis. Eighteen studies were two-arm trials; four studies were three-arm trials involving two different fixed doses of the combination group and UDCA monotherapy control group; one was a four-arm trial involving three different fixed doses of the combination group and UDCA control group. Figure 3 presents a network diagram of a meta-analysis comparing the efficacy of multiple treatments for improving ALP levels in patients with UDCA-refractory PBC. A Bayesian network meta-analysis was performed using a random-effects model to compare the efficacy of different combined treatment regimens in reducing the average ALP level in UDCA-refractory PBC.

**Table 2:** Characteristics of included studies

Study	Design	Treatment/control	Dose	ALP baseline levels (U/L)	Duration	Size
Corpechot 2018	RCT	BEF plus UDCA/UDCA	BEF: 400 mg per day; UDCA: 900–1500 mg per day	244/242	24 months	100
Hosonuma 2015	RCT	BEF plus UDCA/UDCA	BEF: 400 mg per day; UDCA: 600–900 mg per day	423/454	96 months	27
Takeuchi 2011	RCT	BEF plus UDCA/UDCA	BEF: 400 mg per day; UDCA: 600 mg per day	411/602	24 months	37
Iwasaki 2008	RCT	BEF plus UDCA/UDCA	BEF: 400 mg per day; UDCA: 600 mg per day	643/608	12 months	22
Itakura 2004	RCT	BEF plus UDCA/UDCA	BEF: 400 mg per day; UDCA: 600 mg per day	618/463	6 months	16
Kanda 2003	RCT	BEF plus UDCA/UDCA	BEF: 400 mg per day; UDCA: 600 mg per day	700/550	6 months	22
Nakai 2000	RCT	BEF plus UDCA/UDCA	BEF: 400 mg per day; UDCA: 600 mg per day	NA	12 months	23
Mayo 2018	RCT	Aldafermin plus UDCA/UDCA	NGM 282:0.3 mg or 3 mg per day; UDCA: 15 mg/kg per day	244/242	1 month	45
Nevens 2016	RCT	OCA plus UDCA/UDCA	OCA: 5 mg or 10 mg or per day; UDCA: 13–15 mg/kg per day	423/454	12 months	217
Hirschfield 2015	RCT	OCA plus UDCA/UDCA	OCA: 10 mg or 25 mg or 50 mg per day; UDCA: 15.6–16.3 mg/kg per day	294.4/290/286.9/275.2	3 months	165
Kjærgaard 2021	RCT	OCA + UDCA/UDCA	OCA: 5 mg or 10 mg per day; UDCA: Not mentioned	216	3 months	8
Cheung 2016	CS	FEF + UDCA/UDCA	FEF:200 mg per day; UDCA: 13–15 mg/kg per day	302/318	11 months	120
Liberopoulos 2010	CCS	FEF + UDCA/UDCA	FEF: 200 mg per day; UDCA: 600 mg per day	195/232	2 months	10
Li 2022	RCT	FEF + UDCA/UDCA	FEF: 200 mg per day; UDCA: 13–15 mg/kg per day	189.2/217.96	3 months	48
Wang 2022	CS	FEF + UDCA/UDCA	FEF: 200 mg per day; UDCA: 13–15 mg/kg per day	321.3/333.08	12 months	106
Dohmen 2013	CCS	BEF + UDCA/FEF + UDCA	BEF: 400 mg per day; FEF: 80 mg per day;	595.9/522.5	12 months	21
Hirschfield 2021	RCT	Budesonide + UDCA/UDCA	Budesonide: 9 mg per day; UDCA: 12–16 mg/kg per day	262/256	36 months	62
Vuppalanchi 2022	RCT	Saroglitazar + UDCA/UDCA	Saroglitazar: 2 mg or 4 mg per day UDCA: 13 mg/kg per day	351.3/323.2/294.9	4 months	37
Schattenberg 2021	RCT	Elafibranor + UDCA/UDCA	Elafifibranor: 80 mg or 120 mg per day UDCA: 13–15 mg/kg per day	350.6/263.7/296.2	3 months	45
Kowdley 2023	RCT	Elafibranor + UDCA/UDCA	Elafifibranor: 80 mg per day UDCA:13–15 mg/kg per day	321.3/ 321.9	13 months	161
Ding 2022	CS	FEF + UDCA/UDCA	FEF: 200 mg per day; UDCA: 13–15 mg/kg per day	328.9/309.4	12 months	59
Ding 2023	CS	FEF + UDCA/UDCA	FEF: 200 mg per day; UDCA: 13–15 mg/kg per day	365.3/330.2	12 months	118
Hirschfield 2023	RCT	Seladelpar + UDCA/UDCA	Seladelpar: 5 mg or 10 mg per day UDCA: 13–15 mg/kg per day	290.5/290.8/293.4	3 months	265

*RCT* Randomized controlled trial, *CS* Cohort study, *CCS* Case–control study, *UDCA* Ursodeoxycholic acid, *OCA* Obeticholic acid, *BEF* Bezafibrate, *FEF* Fenofibrate, *ALP* Alkaline phosphatase, *NA* Not available

### Comparison between traditional pairwise and network meta-analyses

Table 3 shows the results of traditional pairwise and network meta-analyses. Overall, statistical heterogeneity was moderate to severe. Although the pooled estimates showed small differences, the confidence intervals from the traditional pairwise meta-analyses and the credibility intervals from the Bayesian network meta-analyses are, in general consistent. Confidence intervals for traditional pairwise meta-analyses and credibility intervals for Bayesian network meta-analyses not overlapping 0 were considered statistically significant. There was no significant difference in efficacy between different doses of the same drug.

**Table 3:** Assessment of heterogeneity for direct comparisons and comparison of outcomes between pair-wise meta-analysis and network meta-analysis

Treatment comparisons	Results of pair-wise meta-analysis	*I*^2^(%)	Results of network meta-analysis
Bezafibrate + UDCA/UDCA	142.52(72.09, 212.96)	75.7%	104.49 (60.41, 161.92)
Fenofibrate + UDCA/UDCA	79.18(63.05, 95.30)	92.4%	87.81 (52.34, 129.79)
Bezafibrate + UDCA/fenofibrate + UDCA	72.2(− 5.33, 149.73)	NA	15.76 (− 36.77, 80.70)
Budesonide + UDCA/UDCA	86(− 4.09, 176.09)	NA	84.59 (− 39.94, 210.26)
OCA 10 mg + UDCA/UDCA	66.45(39.51, 93.39)	70.5%	65.21 (8.99, 121.80)
OCA 25 mg + UDCA/UDCA	64.24(55.44, 73.04)	NA	NA
OCA 50 mg + UDCA/UDCA	51.99(45.09, 58.89)	NA	NA
OCA 10 mg + UDCA/OCA 25 mg + UDCA	1.84(− 12.53, 16.21)	NA	NA
OCA 10 mg + UDCA/OCA 50 mg + UDCA	10.41 (− 2.88, 23.7)	NA	NA
OCA 25 mg + UDCA/OCA 50 mg + UDCA	12.25 (1.16, 23.34)	NA	NA
Aldafermin 0.3 mg + UDCA/UDCA	54.3(12.46, 96.14)	NA	NA
Aldafermin 3 mg + UDCA/UDCA	69.3(24.02, 114.58)	NA	69.72 (− 33.18, 170.67)
Aldafermin 0.3 mg + UDCA/NGM282 3mg + UDCA	− 15 (− 61.05, 31.05)	NA	NA
Saroglitazar 2 mg + UDCA/UDCA	131.56(7.81,256.92)	NA	132.09 (13.99, 247.04)
Saroglitazar 4 mg + UDCA/UDCA	151.58 (144.01, 159.15)	NA	NA
Saroglitazar 2 mg + UDCA/Saroglitazar 4 mg + UDCA	− 9.12(− 17.4, − 0.84,)	NA	NA
Elafibranor 80 mg + UDCA/UDCA	142.99(77.38, 208.59)	93.1%	140.73 (74.34, 209.98)
Elafibranor 120 mg + UDCA/UDCA	116.6 (84.46, 148.74)	NA	NA
Elafibranor 80 mg + UDCA/Elafibranor 120 mg + UDCA	62.2(27.15,97.25)	NA	NA
Seladelpar 5 mg + UDCA/UDCA	92.85(61.76,123.94)	NA	NA
Seladelpar 10 mg + UDCA/UDCA	117.67(85.86,149.48)	NA	117.39 (19.97, 213.95)
Seladelpar 5 mg + UDCA/Seladelpar 10 mg + UDCA	− 24.82(− 56.16, 6.52)	NA	NA

*UDCA* Ursodeoxycholic acid, *OCA* Obeticholic acid, *NA* Not available

### Results from the network meta-analysis

**Fig. 4:**
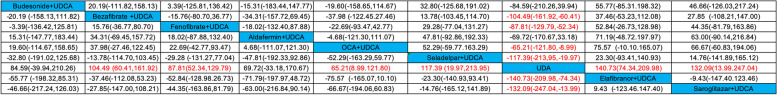
Efficacy of all treatments according to network meta-analysis

Figure 4 illustrates the MD for clinical improvement in ALP levels with 95% credibility intervals obtained from the indirect comparisons of the included regimens. As shown in the figure, 95% credibility intervals (CrI) not overlapping 0 were considered statistically significant. Significant results are marked in red. The combination treatments showed a trend in reducing the levels of ALP more effectively when compared with UDAC monotherapy in patients with UDCA-refractory PBC. In terms of improving ALP biochemical levels, bezafibrate combined with UDCA (MD 104.49, 95% CI 60.41, 161.92), fenofibrate combined with UDCA (MD 87.81, 95% CI (52.34, 129.79), OCA combined with UDCA (MD 65.21, 95% CI 8.99, 121.80), seladelpar combined with UDCA (MD 117.39, 95% CI 19.97, 213.95), elafibranor combined with UDCA (MD 140.73, 95% CI 74.34, 209.98), saroglitazar combined with UDCA (MD 132.09, 95% CI 13.99, 247.04) was more effective than UDCA monotherapy, and the difference was statistically significant.

**Fig. 5:**
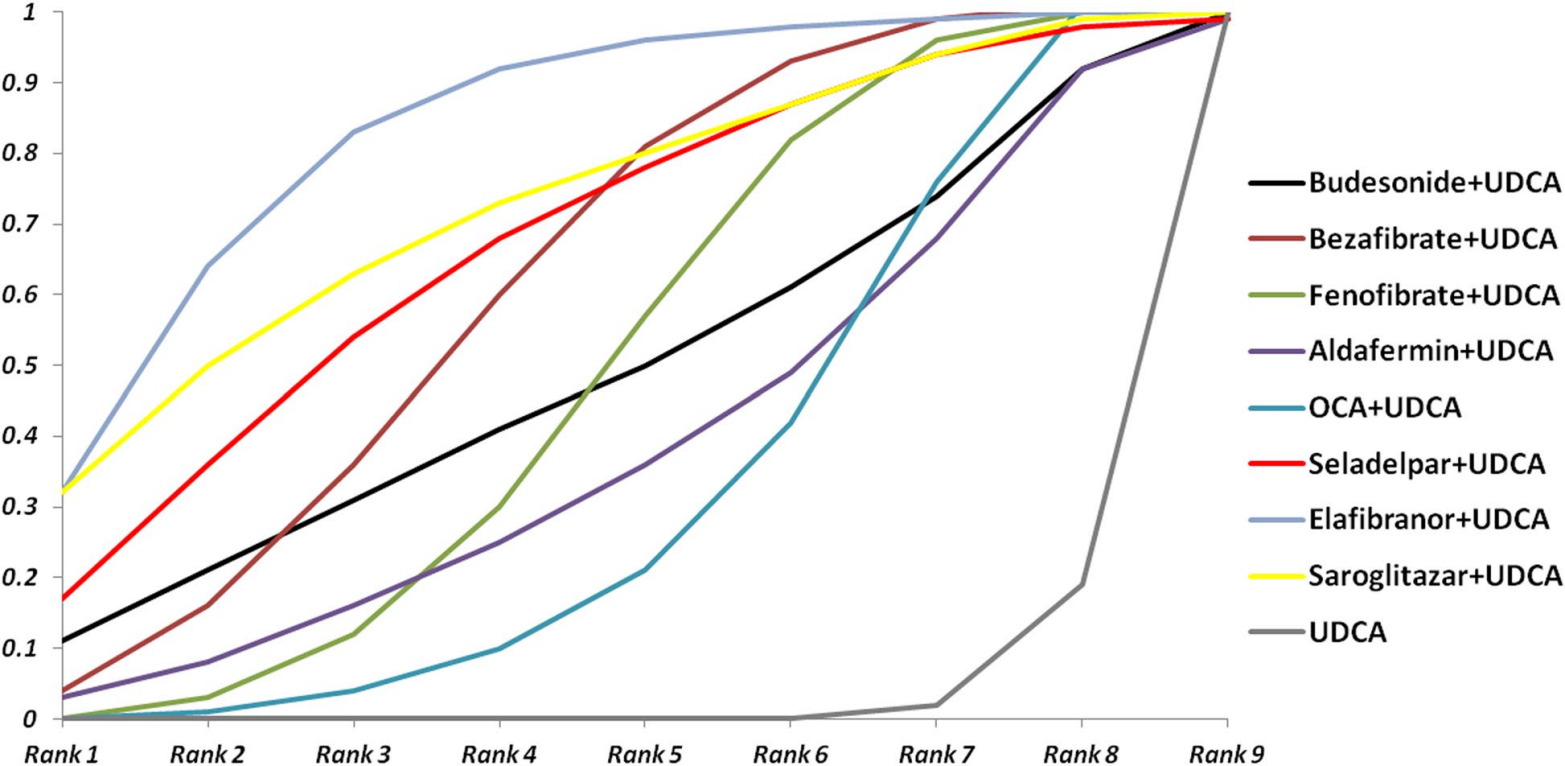
Cumulative probability graph

The cumulative probability graph showed the likelihood of each intervention being the optimal treatment option, with a larger area under the curve indicating a greater likelihood of the intervention being the optimal treatment option. Figure 5 shows the cumulative probability of each treatment being the optimal treatment regimen for improving ALP biochemical levels. Elafibranor in combination with UDCA was the most likely (32%) to be the optimal drug regimen for improving ALP biochemical levels, followed by saroglitazar in combination with UDAC (18%), then bezafibrate in combination with UDAC (20%) and so on. Cumulative probability of eafibranor and saroglitazar were quite close.

### Consistency and convergence analysis

All PSRF values of the different parameters were limited to 1, and it demonstrated that this research achieved good convergence efficiency. The result of the consistency model was reliable. In general, the *P* values were all greater than 0.05, indicating that there was no significant difference between the results of the direct and indirect comparison (Table 4), and no obvious inconsistency within the networks for any of the three outcomes was found in the node-splitting analysis.

**Table 4:** Results of node-splitting analysis

Comparison	*p* value
bezafibrate + UDCA/fenofibrate + UDCA	0.08
bezafibrate + UDCA/UDCA	0.07
fenofibrate + UDCA/UDCA	0.08

### Sensitivity analysis

The network meta-analysis compared the differences in efficacy between different interventions by pooling estimates of the differences in ALP levels before and after treatment with different interventions. Differences in the baseline levels of participants involved in different interventions in the included studies may affect the reliability of our meta-analysis results. Table 2 summarizes the ALP baseline levels of all participants in the study. The data was presented as an average value, with the treatment group in the front and the control group in the back. The multiarm study presented the mean value according to the doses of the drug in the treatment group from low to high. We found that participants in some studies involving bezafibrate in combination with UDAC had significantly higher ALP baseline levels than participants in other interventions, which may exaggerate the efficacy of the combination regimen of bezafibrate and UDAC. We performed a secondary analysis to assess the stability of the results by excluding studies in which participants’ ALP baseline levels differed significantly from other studies. We excluded five studies [13, 42–44, 50] for sensitivity analysis, and the results showed that elafibranor in combination with UDCA was the most likely (35%) to be the optimal drug regimens for improving ALP biochemical levels, followed by saroglitazar in combination with UDAC (21%). Differences in study design may lead to potential heterogeneity to a certain extent. We also excluded six non-RCT studies [10, 45, 46, 48–50] for secondary analysis, and the results showed that saroglitazar in combination with UDCA was the most likely (24%) to be the optimal drug regimen for improving ALP biochemical levels, followed by elafibranor in combination with UDAC (23%). Sensitivity analyses did not change the ranking of the top two drug regimens, and the cumulative probability of the optimal and suboptimal drug regimens was always extremely close. Seladelpar showed better efficacy than bezafibrate in sensitivity analyses. It is worth noting that all these drugs are PPAR agonists without exception. When we excluded both studies with large differences in participants’ ALP baseline levels and non-randomized controlled studies, consistently, the results closely resembled our primary analysis in the sensitivity analysis. This suggested that the results of our network meta-analysis are robust relatively.

### Publication bias

**Fig. 6:**
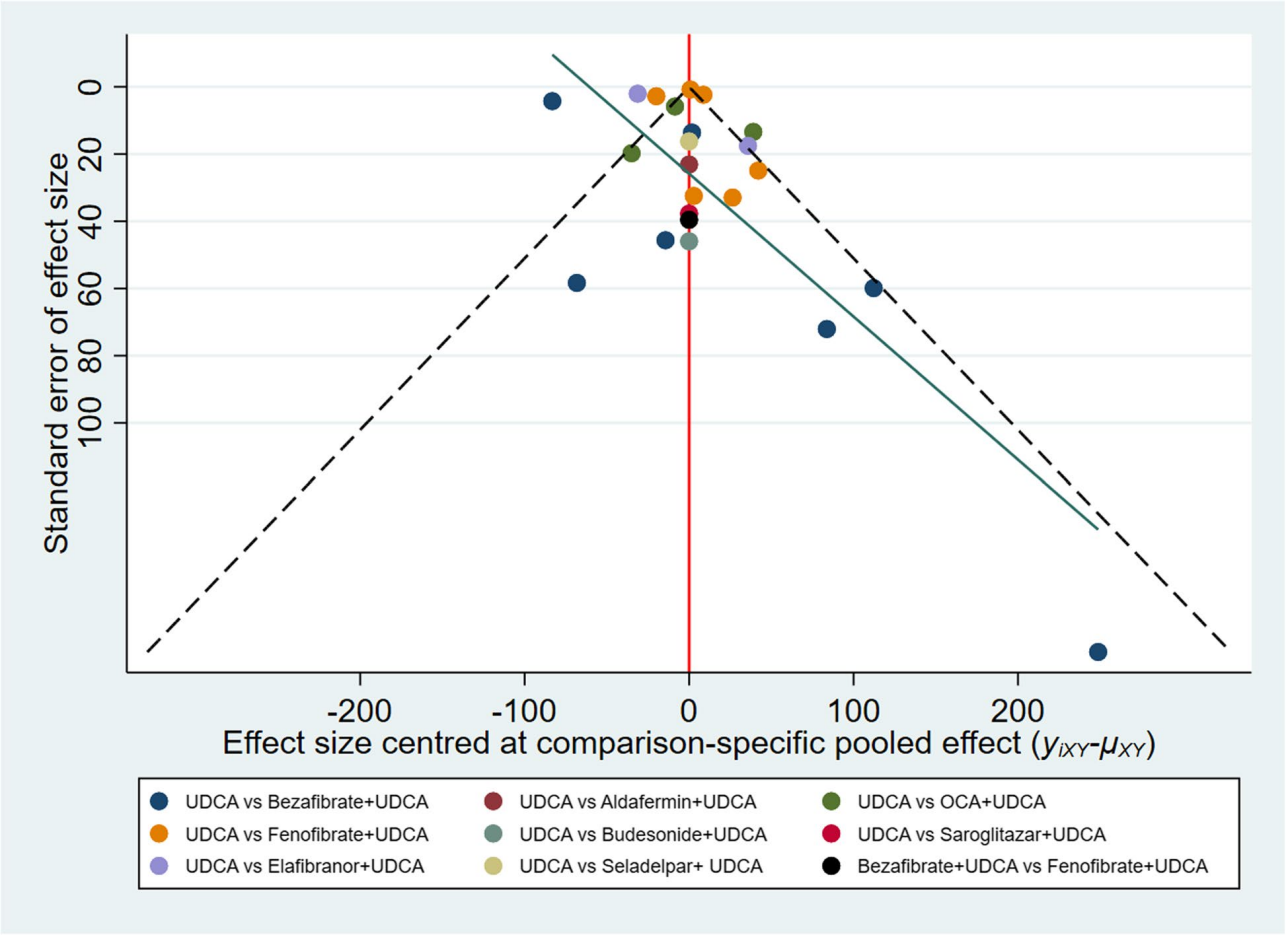
Funnel plots showing the risk of publication bias in the meta-analysis

A funnel plot was used to evaluate the presence of publication bias. As shown in Fig. 6. The graph is relatively symmetric. Visual inspection of the funnel plots did not lead to concerns about publication bias.

## Discussion

In this network meta-analysis, we comprehensively reviewed the efficacy of different interventions in patients with UDCA-refractory PBC.

UDCA has been the mainstay of therapy in patients with primary biliary cholangitis for more than 20 years [54], but up to 40% of patients had an inadequate response to UDCA monotherapy in this clinical background [6]. Published studies have shown that the development of cirrhosis and higher mortality in patients with PBC are strongly associated with a lack of biochemical response to UDCA [55]. Liver biochemistry, such as alkaline phosphatase and total bilirubin, can predict the clinical outcomes of patients with PBC and are now considered surrogate endpoints in therapy trials [56]. The current consensus is that ALP and total bilirubin levels are the two most important parameters for assessing treatment response [57]. Based on this clinical background, we performed this network meta-analysis to compare the effect of different therapy regimens for improving ALP levels in patients with PBC refractory to UDCA. As shown in our network meta-analysis, elafibranor at a dose of 80 mg showed the best efficacy in improving PBC patients refractory to UDCA, followed by saroglitazar at a dose of 4 mg. In view of the fact that both elafibranor and saroglitazar are phase II randomized controlled studies so far while related phase III randomized controlled studies are ongoing, this result needs to be interpreted with caution, and the results of our network meta-analysis need to be further validated by large scale, high-quality phase III studies in the future. It is worth noting that further studies are underway at a daily dose of 2 mg and 1 mg due to the higher incidence of elevated liver enzymes observed with the 4 mg dose [18]. Peroxisome proliferator-activated receptor (PPAR) plays a central role in the anti-cholestatic effects [58]. In terms of affecting hepatic biochemical parameters, peroxisome proliferator-activated receptor (PPAR) agonists manifest significant effects in reducing ALP levels but may be associated with elevated transaminase concentration levels [28]. As shown in our analysis, four PPAR agonists were more effective than other drugs in reducing ALP biochemical levels as adjunctive therapy for patients with PBC refractory to UDCA, bezafibrate and fenofibrate were second only to elafibranor and saroglitazar. Dohmen et al. [50] found that in PBC patients refractory to UDCA, the effect of bezafibrate in decreasing the ALP levels was slightly superior to that of fenofibrate, which was generally consistent with the results of our network meta-analysis although not statistically significantly.In view of the fact that this is a small sample study, the reliability of conclusions needs to be further confirmed by high-quality large-size studies of the future.

The results of our network meta-analysis suggested that the effect of OCA was inferior to four PPAR agonists while superior to other drugs in improving ALP levels in PBC patients refractory to UDCA. The results of a multicenter observational study by Reig et al. [59] also showed that fibrates had a greater reduction in ALP while OCA had a greater reduction in alanine aminotransferase as second-line therapy in PBC refractory to UDCA. OCA had been shown to be effective in decreasing ALP levels when added to UDCA in patients with PBC refractory to UDCA in several randomized double-blinded, placebo-controlled trials. Therefore, it is the only other medication to be FDA-approved for the treatment of PBC. OCA is effective at considerably lower doses; currently, the recommended dose of OCA is 5 mg or 10 mg. Despite there being an incremental benefit observed with adjustment to the higher dose, OCA can cause worsening pruritus in the meanwhile, which was a key symptom of primary biliary cholangitis [24–26]. Our network meta-analysis also failed to reveal statistically significant differences in efficacy between different doses of the same drug. In the absence of UDCA, OCA monotherapy also could significantly improve ALP and other liver biochemical parameters levels [26]. However, compared with other therapy regimens, budesonide, and aldafermin seemed to be less effective in improving ALP levels in patients with PBC refractory to UDCA as adjunctive therapy as shown in our analysis.

We aim to minimize the possibility of the generation of heterogeneity by distinguishing drug groups with different doses in our network meta-analysis. Therefore, on the premise that only analyzing the efficacy of drugs without analyzing the safety profile, the results of our analysis were only used to identify the best therapy regimen to improve ALP levels but not as the basis of the best-recommended doses of a drug.

It is worth noting that all included drug regimens were effective in improving ALP levels in PBC patients as observed in the included RCTs. In our network meta-analysis, no statistically significant differences were found between the included combination regimens. Rank probability plots only suggest the most likely optimal drug regimens. The fact that drugs with different receptors and mechanisms could improve the ALP levels may support the application of multiple combination therapies (e.g., UDCA + PPAR + FXR) in PBC refractory to UDCA. The study by Smets et al. [60] showed that triple therapy with UDCA, OCA, and bezafibrate further improved biochemical markers of cholestatic liver injury and reduced pruritus severity in patients with PBC refractory to a combination of UDCA and OCA. The study by Sore et al. [61] showed that in patients with UDCA-refractory PBC who failed to achieve a good clinical response by receiving OCA or fibrates as second-line therapy, triple therapy with UDCA, OCA, and fibrate was able to normalize liver biochemistry and improves pruritus. Primary biliary cholangitis has features of multiple autoimmune diseases, so immunosuppressants may have important value in the treatment of patients with primary biliary cholangitis. Despite rituximab has shown promise in autoantibody-related immune-mediated diseases, its biochemical efficacy in patients with PBC with an incomplete response to UDCA is limited [62]. A published study showed that compared to UDCA monotherapy, triple therapy with UDCA, prednisone, and azathioprine has additional beneficial effects on symptoms, biochemical and histological parameters in patients with UDCA-refractory PBC [63], and superior clinical benefit was also observed in PBC patients with high levels of IgG and transaminases [64]. This provides a factual basis for the design of research programs in the future. These results support the evaluation of triple therapy in future large-sample, long-term controlled trials and the further exploration of the optimal treatment regimen for patients with UDCA-refractory PBC.

Our study has several strengths. To the best of our knowledge, this is the first network meta-analysis making an attempt to compare the efficacy of different drugs as adjunctive therapy in patients with PBC who respond incompletely to UDCA monotherapy. Having conducted a rigorous and extensive literature search, we are confident that all relevant clinical trials have been properly identified. Moreover, a random-effects model was used to pool the most fully adjusted estimates to reduce the confounding bias in the results to the utmost extent.

We have to admit that there are some limitations of our meta-analysis. Firstly, the sample sizes were overall small in most included studies in this analysis. Due to the lack of sufficient available phase III randomized controlled trials for analysis, the included studies involving elafibranor, saroglitazar, and aldafermin were all phase II randomized controlled trials with small samples, as a consequence, the large effect size may have affected the results. And a part of the studies is observational studies, potential heterogeneity caused by differences in study design is unavoidable accordingly. Currently, phase III randomized controlled trials of elafibranor, saroglitazar, seladelpar, and aldafermin are ongoing, and results are pending. Secondly, we did not conduct subgroup analysis in terms of the duration of different medications since there is not enough available data, so potential heterogeneity to a certain extent was inevitable. However, by reviewing the full text, we found that the duration of treatment was longer than 3 months in most included studies, and the levels of ALP in patients tended to be stable after that. Finally, we only analyzed the efficacy of the drug without analyzing its safety because factors such as inconsistency in the definition of adverse events in different studies and no adverse events observed in some studies with small sample sizes could greatly affect the reliability of the results.

In conclusion, for PBC patients with incomplete response to UDCA monotherapy, the combination regimen can have better clinical benefits than UDCA monotherapy. Our study found that on the basis of UDAC treatment, PPAR agonists as second-line therapy were more effective than any other drugs with other mechanisms in improving ALP biochemical levels, with elafibranor being the best, followed by saroglitazar. However, due to the lack of sufficient randomized controlled studies for some drugs included in our studies, the reliability of the conclusions needs to be further confirmed by future high-quality large-sample phase III randomized controlled studies.

## Data Availability

All data generated or analyzed during this study are included in this manuscript.
